# CD8^+^ T Cells: GITR Matters

**DOI:** 10.1100/2012/308265

**Published:** 2012-04-30

**Authors:** Simona Ronchetti, Giuseppe Nocentini, Maria Grazia Petrillo, Carlo Riccardi

**Affiliations:** Dipartimento di Medicina Clinica e Sperimentale, Sezione di Farmacologia, Tossicologia e Chemioterapia, Università di Perugia, Via del Giochetto, 06100 Perugia, Italy

## Abstract

As many members of the tumor necrosis factor receptor superfamily, glucocorticoid-induced TNFR-related gene (GITR) plays multiple roles mostly in the cells of immune system. CD8^+^ T cells are key players in the immunity against viruses and tumors, and GITR has been demonstrated to be an essential molecule for these cells to mount an immune response. The aim of this paper is to focus on GITR function in CD8^+^ cells, paying particular attention to numerous and recent studies that suggest its crucial role in mouse disease models.

## 1. Introduction

Known as an important costimulatory molecule in all T-cell subsets, glucocorticoid-induced TNFR-related gene (GITR), a member of the TNFR superfamily, is considered a key regulator in a multitude of immune functions and in some tissues [1, 2]. GITR is expressed and further upregulated on most immune cell types like T regulatory cells (Tregs), naïve T cells, natural killer cells (NKs), and at low levels in B cells, macrophages, and dendritic cells [3, 4]. Different splicings of GITR gene have been identified, including a soluble form [5]. GITR's role has been studied in a number of physiological conditions and cells like keratinocytes [6], bone [7], sympathetic neuron development [8], bone marrow stromal cells [9], microglia [10], and in a variety of autoimmune/inflammatory pathologies in murine models. Such studies reveal GITR as a pivotal mediator in inflammation processes and autoimmune diseases as described in murine experimental colitis [11, 12], acute and chronic inflammation of the lung [13, 14], collagen-induced arthritis [15], splanchnic artery occlusion (SAO) shock [16], thyroiditis [17], experimental autoimmune encephalomyelitis [18], acute pancreatitis [19], and multiple organ dysfunction syndrome (MODS) [20]. Despite their name, glucocorticoids are unnecessary for GITR upregulation [21], unlike demonstrated for another glucocorticoid-induced gene [22, 23]. GITR-derived signals promote an inflammatory environment as indicated by the attenuated course taken by GITR^−/−^ mice during the aforementioned autoimmune/inflammatory experimental diseases.

GITR is triggered by its ligand (GITRL), mainly expressed in antigen-presenting cells and endothelial cells [24, 25]. The costimulatory effect of GITR triggering in T cells, both conventional CD4^+^ and CD8^+^ cells, causes enhanced T-cell expansion and cytokine production [26–30]. Conversely, GITR engagement in NK cells induces an inhibitory effect [31–33], even though a separate study provides opposite results [34]. Costimulation by GITR is also found either to activate [35] or to inhibit NKT cells [36]. The role played by GITR in Tregs appears to be more complex. When it was found highly expressed in Treg cells, GITR appeared to abrogate Treg-mediated suppression, when triggered by an anti-GITR mAb [37, 38]. However, one later study suggested that strong co-activation of effector T cells was responsible for this effect, since GITR-triggered effectors were found to be resistant to Treg-mediated suppression [39]. Although GITR influences Treg function, it does not take part to the mechanism of suppression, since we found that GITR-KO Treg cells are able to suppress as well [26]. Furthermore, an anti-GITR treatment in mouse tumor models alters the number of tumor infiltrating Treg cells [40], and GITRL transgenic mice show an increased absolute number of T regulatory cells [41]. So there has been confusion about the actual function of GITR on Treg cells. Currently, the most accepted explanation about GITR function in Treg and T effector cells is that GITR engagement activates both cells thereby causing resistance of effector cells to Treg suppression, inhibition of Treg cell activity and Treg expansion [4, 26, 42–44]. Another piece of the puzzling function of GITR in Treg cells has been recently added by the discovery of a human CD4^+^ subpopulation with regulatory activity that expresses GITR and CD127 but only low levels of CD25, so that GITR can now be considered as a marker of these cells [45, 46].

Recent works have found a correlation between GITR and some human pathologies: in the pathogenesis of rheumatoid arthritis (RA), the expression of GITR on macrophages in human RA synovium may enhance inflammatory activation of these cells [47]; in atopic dermatitis, the interaction of GITR with its cognate ligand, GITRL, may perpetuate local inflammation [48]; finally, one polymorphism of GITR gene seems to be associated with Hashimoto's disease prognosis [49]. A separate issue deals with the relationship of GITR and tumors, well reviewed by Placke et al. [50] and Schaer et al. [44], who describe how GITR importance has grown up since it was found to be involved in tumor rejection, in studies that used anti-GITR antibodies or GITR recombinant proteins, as also described below in this paper. Accordingly, GITR expression in tumor infiltrating lymphocytes (TILs) has been found to be associated with cancer progression in patients suffering from esophageal adenocarcinomas. Although studies in mice and men could lead to contrasting conclusions about the exact role of GITR in the same cell type, many efforts are being made to transfer the knowledge of GITR function to the clinics. The aim is the application of tools like anti-GITR mAbs or recombinant proteins like GITR-Fc to therapy of cancer and infectious or inflammatory diseases.

This paper focuses on the role of GITR in the powerful modulation of CD8^+^ T-cell function, a field that still needs more investigation owing to the pivotal role played by CD8^+^ cells in cancer rejection and infectious diseases.

## 2. Expression of GITR in CD8^+^ T Cells

Despite the role of GITR has been poorly investigated in CD8^+^ cells as compared to the wide range of studies in CD4^+^ cells, a picture of GITR expression and function in CD8^+^ cells begins to be elucidated. Mouse CD8^+^ T cells express GITR at basal level and upregulate GITR after activation [26, 27, 51–53], while human CD8^+^ cells show GITR expression only after activation [54, 55]. In a study aimed at identifying molecular markers of human CD8^+^CD28^−^ cells, GITR is found to be one of the expressed genes in primed cells [55], confirming that no GITR can be detected at the steady state in human CD8^+^ cells, either in CTLs or in peripheral regulatory T cells. Only a regulatory population of human thymic CD8^+^CD25^+^ cells spontaneously expresses GITR. In a pathologic condition such as viral infection, in vivo kinetics of GITR expression shows its increase on antigen-specific CD8^+^ T cells after ocular HSV infection in the draining lymph nodes, suggesting an involvement of GITR/GITRL system in the regulation of virus-induced immuno-inflammatory lesions [56]. Interestingly, GITR expression is found to be enhanced in CD8^+^ tumor-specific cells thus suggesting an involvement of GITR in CTL-mediated tumor rejection (see below).

## 3. The Major Role of GITR in CD8^+^ Cells

The unquestioned role of GITR as a costimulatory molecule in T cells has been deeply investigated over the last years and has yielded a flourishing literature predominantly dealing with either conventional or Treg CD4^+^ cells [2, 4, 50]. However, the importance of GITR in CD8^+^ cells has been established as well by ex vivo and in vivo studies as described below. We have demonstrated that the costimulatory role of GITR is indispensable to fully activate CD8^+^ cells since its absence impairs the proliferation response of these cells to CD28 costimulation [51]. Conversely, CD28^−/−^ CD8^+^ cells can be fully activated by GITR costimulation. Interestingly, no differences are observed in CD4^+^ cells between GITR^−/−^ and control mice, suggesting a specific role for GITR-mediated costimulatory signals in CD8^+^ cells. As a consequence, NF-*κ*B, one of the best characterized mediator of GITR signalling pathway, is impaired in CD28-costimulated GITR^−/−^ CD8^+^ cells, indicating GITR as a unique costimulatory molecule independent of CD28 [51]. To support the peculiar role of GITR in CD8^+^ cells, two brilliant works have previously demonstrated a separate function of GITR in CD4^+^ and CD8^+^ cells. Muriglan et al. show that GITR stimulation by an anti-GITR mAb (DTA-1) enhances alloreactive CD8^+^CD25^−^ T-cell proliferation increasing GVHD and exerting opposite effects in alloreactive CD4^+^CD25^−^ cells [52]. Moreover, Kim et al. show that the engagement of GITR by the same antibody DTA-1 regulates the ability of donor CD8^+^ cells to respond while they are in the process to become tolerant, pushing the shift from chronic GVHD to acute GVHD [53]. Therefore, dissecting the immunological mechanisms that determine the development of GVHD holds therapeutic promise for manipulation of the GITR/GITRL system in the prevention of GVHD and other related pathologies.

## 4. GITR Function in Viral Infections

A few in vivo studies evidence how GITR signals may be important for antiviral activity of CD8^+^ cells. In a mouse model of HSV-1 infection, La and coworkers found that treatment with anti-GITR agonistic antibody (DTA-1) causes an expansion of both CD4^+^ and CD8^+^ cells in the draining lymph nodes, with a higher increased number of CD8^+^ cells. Notably, antigen-specific IFN-*γ*-secreting CD8^+^ cells are increased, suggesting GITR stimulation as an important mediator of virus clearance by the immune response [57]. The increase in T-cell responses and the reduction in viral load are also observed when persistently infected mice with Friend virus are treated with anti-GITR therapy combined with adoptive transfer of CD8^+^ cells [58]. In another study by Suvas et al., the same anti-GITR antibody DTA-1 enhances virus-specific T-cell responses mediated by CD4^+^ and CD8^+^. In particular there is an increase of in vivo CD8^+^ T-cell cytotoxicity, as demonstrated [56] by rise in Granzyme B levels in a mouse model of ocular HSV infection, while a decrease of proinflammatory responses of CD11b^+^ cells. The role of GITR stimulation for the expansion of CD8^+^ cells is further and definitely demonstrated by Snell et al. who use GITR^−/−^ OT-I transgenic mice in an in vivo model of severe influenza infection in which purified CD8^+^ GITR^−/−^ OT-I cells are transferred into recipient mice successively infected with influenza A/HK-X31-OVA [59]. They find that GITR is critical for CD8^+^ expansion in both the primary and secondary response to severe influenza virus infection and is required for CD8^+^ survival. Of note anti-GITR DTA-1 antibody induces an enhanced expression of BcL-x_L_, a prosurvival molecule downstream of NF-*κ*B, previously shown to be important in CD8^+^ cells in GITR-sufficient mice [51].

**Figure 1:**
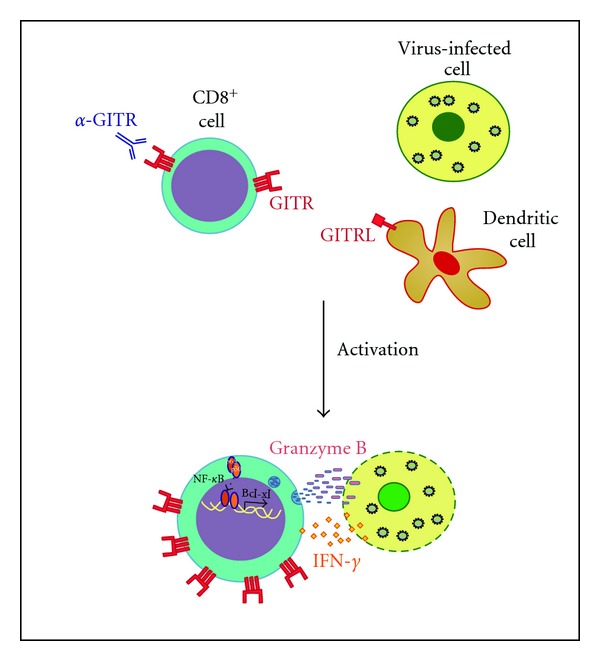
GITR function in antiviral immunity. Treatment with anti-GITR antibody induces activation of CD8^+^ cells through upregulation of GITR, release of IFN-*γ*, Granzyme B, and nuclear translocation of NF-*κ*B with subsequent transcription of target genes. GITRL is expressed in dendritic cells.

Overall, all studies frame GITR as a pivotal molecule in CD8^+^ cells, which seems to contribute to viral clearance and mouse survival in infections in which CD8^+^ cells play a critical role (Figure 1). The only data in contrast with the aforementioned studies are obtained analyzing GITRL transgenic mice, which overexpress GITRL transgene in B cells, where CD8^+^ cells are not expanded while both effector and regulatory CD4^+^ cell numbers are increased [41]. This discrepancy can be explained either with a lack of interaction between CD8^+^ T cells and B lymphocytes or with the fact that GITR is already stimulated at maximum levels in WT mice so that a GITRL increase does not influence the CD8^+^ response.

## 5. GITR, CD8^+^ Cells, and Antitumor Immunity

Several studies have recently revealed a strong tumor rejection potential for both the anti-GITR antibody DTA-1 and the recombinant protein Fc-GITRL [1, 44]. In the attempt to find which cells are involved in such a mechanism, some studies focused their attention on CD8^+^ cells. Although CD4^+^ cells play a role in mediating anti-GITR-induced immune activation against tumors [60, 61], CD8^+^ cells are indispensable for tumor rejection [60–64]. Studies of T-cell depletion effectively demonstrate the major role for CD8^+^ cells. CD8^+^ depletion, in combination with Fc-mGITRL treatment, significantly lowers tumor regression as compared to Fc-mGITRL alone in tumor-bearing BALB/c mice [63]. In contrast, CD4^+^ depletion in combination with Fc-mGITRL treatment results in the same percent tumor regression as CD4^+^ depletion alone. Thus, in Fc-mGITRL immunotherapy, CD8^+^ cells play a critical role [63]. In another study, depletion of CD8^+^ cells in mice injected with GITRL-expressing tumor cells promotes tumor growth [65]. Furthermore, Nishikawa et al. demonstrate that GITRL inhibits CMS5a tumor growth in mice immunized with a CTL epitope and that depletion of CD8^+^ cells blocks the effect of coadministered GITRL [66]. Finally, Côte et al. show that depleting CD8^+^ cells together with the administration of anti-GITR Ab, at the time of inducing the primary tumor, significantly reduces the number of mice rejecting secondary tumors [67]. Therefore, T-cell-specific depletion studies invariably show that GITR stimulation, either by anti-GITR antibodies or recombinant GITRL proteins, activates CD8^+^ response against tumor, which appears to be crucial for tumor rejection.

Also studies of T-cell transfer further support CD8^+^ involvement in tumor rejection that seems to be somehow GITR-dependent. The study by Imai et al. shows that adoptively transferred CD8^+^ cells specific for the CMS5 tumor antigen mERK2 in tumor-challenged BALB/c mice induce tumor regression. Such effect is enhanced by the coadministration of anti-GITR DTA-1 mAb [62]. Another study demonstrates that anti-GITR treatment together with CD8^+^ cells in SCID recipient mice is sufficient to reject the tumor, while CD4^+^ transferred cells are not, although CD4^+^ cells play a central role in helping the functions of CD8^+^, NK and B cells [60]. Therefore, the cytolytic activity of CD8^+^ cells induced by GITR engagement is essential for successful tumor eradication.

But how do CD8^+^ cells exert their action? Do they expand? Experiments performed in vivo with various GITRL recombinant proteins and anti-GITR antibodies prove that the presence of the ligand, either inside the tumor or as a soluble molecule, or treatment with anti-GITR antibody augments CD8^+^ expansion and activity. These results confirm that GITR acts as a costimulatory molecule in CD8^+  ^cells. Piao et al. use i.p. inoculated GITRL^+^ tumors to study CD8^+^ cell response and find an enhanced cytotoxicity and number of these cells [65]. Hu et al. use a soluble Fc-mGITRL in two BALB/c tumor models and demonstrate an increased CD8^+^ activity and Granzyme B production between controls and treated mice [63]. Another interesting study by Cho et al. induces ectopic expression of GITRL in tumor that causes accumulation and increase of activity of CD8^+^ cells [68]. Calmels et al. demonstrate that GITRL recombinant protein causes enhanced cell infiltration of both CD4^+^ and CD8^+^ cells once injected into the tumor [61]. GITRL, therefore, seems to be the key molecule to induce activation in CD8^+^ cells, which expand when GITR is triggered by its cognate ligand.

**Figure 2:**
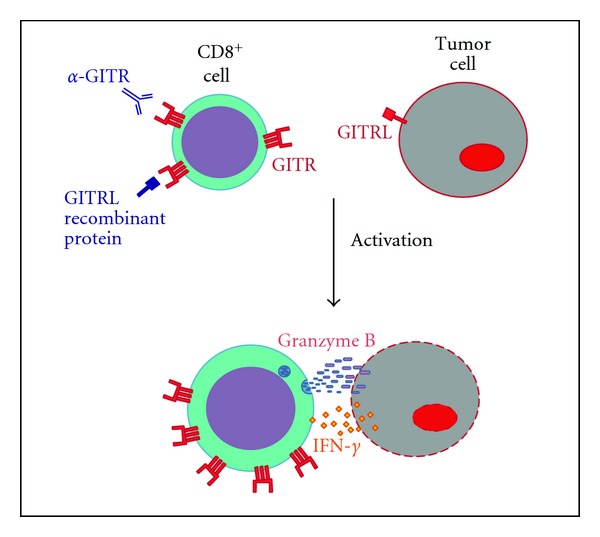
GITR function in antitumoral immunity. Treatment with anti-GITR antibody and GITRL recombinant protein induces activation of CD8^+^ cells through upregulation of GITR, release of IFN-*γ*  and Granzyme B. There is evidence that some tumors express GITRL.

A contribute to antitumor immunity comes from GITRL transfection in dendritic cells, either murine or human, which have been demonstrated to cause expansion and intratumor accumulation of CD8^+^ antigen-specific IFN-*γ*-secreting cells. Once more the forced expression of GITRL enhances the cytolytic and antitumor activity of CD8^+^ cells, which are able to delay tumor growth through the production of IFN-*γ* [69, 70]. Whether GITRL is upregulated in solid tumors is still an open field, since its expression has been found in AML [31] and in some cell lines such as HUVEC EA.Hy926 or EBV-transformed B cell [70], but no study is so far available about GITRL expression in murine or human solid tumors (Figure 2).

When the anti-GITR mAb DTA-1 is used in tumor models, it can activate CD8^+^ and induce tumor rejection as GITRL recombinant proteins do. Mitsui et al. demonstrate that, while CD4^+^ cells provide essential “help” to activate CD8^+^ cells, CD8^+^ are effectively able to kill tumor cells in mice that received injection of a murine colon carcinoma cell line after anti-GITR/anti-CTLA-4 combined treatment. Moreover the number of tumor-infiltrating CD8^+^ cells is augmented, reflecting an enhanced CD8^+^ proliferation [40]. Another work by Cohen et al. studies tumor-specific CD8^+^ cells after adoptive transfer and demonstrates that anti-GITR treatment augments [21] their activation, proliferation, and accumulation in the tumor site leading to a long-term survival [71]. Finally, the work by Côte et al. demonstrates that GITR stimulation with the anti-GITR antibody skews the avidity of polyclonal CD8^+^ cells to tumor-specific Ags. They use a mouse model of melanoma, in which CD8^+^ cells are the major targets of anti-GITR therapy [67]. Such studies demonstrate that an anti-GITR antibody may help fight against tumor cells by stimulating CD8^+^ cells, opening the possibility about the use of these molecules in treatment of cancer also in humans. To this aim, a new clinical trial with an anti-GITR antibody in the therapy of human melanoma gained approval in December 2010 (trial no. NCT01239134). Overall, these studies demonstrate that the use of anti-GITR mAbs and the treatment, or vaccination, with GITRL fusion proteins induce CD8^+^ activation leading either to eradication or reduction of the tumor.

One concern about the use of anti-GITR antibodies is the possibility of development of autoimmunity. Treatments with agonistic anti-GITR Ab may induce or exacerbate autoimmunity through the effect on Treg cells. However, recent works support the demonstration of no overt autoimmunity [40, 72], or to a small extent [64], depending on the route of administration, the anti-GITR dose, the duration of treatment, and the genetic susceptibility of the mouse strain used. However, evidence is accumulating that systemic administration of agonist anti-GITR Ab has a higher probability to induce autoimmunity than local administration. Therefore, in our opinion, studies evaluating strategies to stimulate GITR preferentially in tumors are welcome to facilitate the application to human treatment.

Once CD8^+^ cells are activated by an anti-GITR Ab or its natural ligand, they expand and become activated by increasing IFN-*γ*  production [40, 62, 64, 66, 71–73]. So GITR-derived signals make CD8^+^ lymphocytes able to proliferate, activate, accumulate into the tumor site, and kill tumor cells either alone or with the help of other cytolytic cells such as NK [60]. But how do CD8^+^ cells manage their relationship with Treg tumor infiltrating cells? Are they sensitive or resistant to tumor suppression by Tregs? Tumors contain a large number of infiltrating Treg cells, helping cancer cells to escape immunity. Except one study [71], some others refer about a proven resistance of in vivo GITR-stimulated CD8^+^ cells to tumor Treg suppression [40, 65, 66, 73]. Therefore, it is possible that CD8^+^ cells become locally activated by GITRL^+^ tumors and escape the regulatory function of Treg cells.

## 6. Concluding Remarks

CD8^+^ cells remain the key players in the fight against infections and tumors thanks to their cytotoxic activity [74]. GITR has recently come out as an important molecule for CD8^+^ activity, and targeting GITR in these cells might be a potential instrument for treatment of infections and tumors (Figure 1). Vaccination strategies aimed at stimulating GITR on CD8^+^ cells will represent one of the future possibilities for tumor immunotherapy. The transfer of the knowledge deriving from mouse models to the clinical setting is hampered by the structural differences between murine and human GITR and by the differences in the tools able to activate GITR, as recently summarized [1]. Nonetheless, we believe that the use of biological tools such as GITRL recombinant proteins might be of benefit to clear viruses and in local anticancer therapies.

## 7. Authors Contribution

S. Ronchetti and G. Nocentini equally contributed to the paper.
